# Association of Lifestyle-Related Comorbidities With Periodontitis: A Nationwide Cohort Study in Korea

**DOI:** 10.1097/MD.0000000000001567

**Published:** 2015-09-18

**Authors:** Jae-Hong Lee, Jung-Seok Lee, Jin-Young Park, Jung-Kyu Choi, Dong-Wook Kim, Young-Taek Kim, Seong-Ho Choi

**Affiliations:** From the Department of Periodontology, Research Institute for Periodontal Regeneration, Yonsei University College of Dentistry, Seoul (J-HL, J-SL, J-YP, S-HC); Department of Health Insurance Research (J-KC); Department of Policy Research Affairs (D-WK); and Department of Periodontology, Ilsan Hospital, National Health Insurance Service, Goyang, Korea (Y-TK).

## Abstract

The aim of this study was to determine the association of periodontitis with lifestyle-related comorbidities (LCs) using data in the Korean National Health Insurance Cohort Database from 2002 to 2013.

This was a retrospective study involving a large national cohort with patient samples (representing 2% of the total Korean population) stratified on the basis of sociodemographic information. Using this precisely extracted database, the correlations between LCs (cerebral infarction, angina pectoris, myocardial infarction, hypertension, diabetes mellitus, rheumatoid arthritis, erectile dysfunction, osteoporosis, and obesity) and periodontitis were investigated while adjusting for confounding bias. Univariate and multiple logistic regression analyses were used to evaluate differences in variable factors.

Among a total of 1,025,340 samples, 321,103 (31.3%) cases were diagnosed with periodontitis. Statistically significant associations were found between all LCs except myocardial infarction and periodontitis (*P* < 0.005).

Periodontitis is significantly and positively correlated with LCs (except for myocardial infarction) after adjusting for confounding bias. In particular, lifestyle-related diseases, erectile dysfunction, and osteoporosis seem to be intimately related to periodontitis.

## INTRODUCTION

More than half of the world's population reportedly has periodontal disease, recording sixth highest amongst the prevalence rate of inflammatory diseases.^1,2^ In South Korea, the recent number of patients who are diagnosed and undergoing treatment for periodontitis has increased by 16.7% per annum. In 2013, 22% of the Korean population (representing about 10 million people) covered by the National Health Insurance Service (NHIS) have received periodontitis treatments, and US$ 500 million has been spent on their treatment.^3^

Periodontitis is a major oral disease that is strongly influenced by age.^4,5^ Other sociodemographic factors such as sex, household income, insurance status, health status, and residence area also tend to affect the degree of periodontitis.^1,6^ Lifestyle-related comorbidities (LCs) such as cerebral infarction (CIn), angina pectoris (AP), myocardial infarction (MI), hypertension, diabetes mellitus (DM), rheumatoid arthritis (RA), erectile dysfunction (ED), osteoporosis, and obesity are regarded as risk factors or risk indicators affecting the character or severity of periodontitis, or reversely, periodontitis is considered as the risk factor for comorbid diseases.^7–9^

Cross-sectional and longitudinal epidemiologic studies have reliably established that microbial pathogens are the risk factors for periodontitis, but the relationships between various intrinsic and extrinsic associated factors and periodontitis remain unclear.^10,11^ Small, single studies on the Korean population have been previously performed such as the Korean National Health and Nutrition Examination Survey that examined approximately 10,000 Koreans to identify the relationship between genetic, hematologic, anthropometric, sociodemographic factors and comorbidities; however, a retrospective large national cohort study involving patient samples stratified on the basis of demographic information has not been conducted.^6,12,13^ Therefore, the present study aimed to determine the prevalence and association of periodontitis in relation with LCs using data from the Korean National Health Insurance Cohort Database (KNHICD).

## MATERIALS AND METHODS

### Data Sources and Study Samples

Almost all (97.1%) of the Korean population (51,448,491 people, comprising 25,780,383 males and 25,668,348 females) was covered by the mandatory social NHIS in 2013.^3^ The records in the NHIS database include patients’ sociodemographic information, their use of in- and outpatient services, dental services, and traditional Korean medical services, and pharmacy dispensing claims.

In the present study, a random sample of 1,025,340 people (representing 2% of the total population) insured by the NHIS in 2002 was selected by the NHIS Big Data Steering Department, which is conducting multistage sampling and investigating a representative sample of the whole population for the 11 years from 2002 to 2013. Initial cohort members were selected by stratified sampling of 1,476 strata according to sex (2 groups), age (18 groups: those age 1–79 years in 5-year intervals, and those >80 years), and income level (41 groups: those with area- and job-based health insurance accounting for 20 groups each, and health insurance beneficiaries in 0 group). Members for whom records were no longer available due to death or emigration were excluded from 2003, and these were replaced by members recruited from newborns in the same calendar year. To protect the individual's privacy, each patient's identification number was anonymized. Diseases were diagnosed using the Korean Classification of Disease, sixth edition (KCD-6), which is a modified version of the International Classification of Disease (ICD-10) that is used in Korean National Health Insurance. The retrospective KNHICD of patients with periodontitis diagnosed by a general or specialized dentist was used based on the claims data from 2002 to 2013. We defined periodontitis as the presence of one of the following diagnostic codes as determined after receiving dental treatment: acute periodontitis (K052), chronic periodontitis (K053), periodontosis (K054), other periodontal disease (K055), and unspecified periodontal disease (K056). Inclusion criteria were based on criteria of the American Academy of Periodontology as well as ICD-10 classification criteria.^14,15^

This study was reviewed and approved by the institutional review board of NHIS Ilsan Hospital (approval #2015-06-022).

### Confounding Variables

This study assessed sociodemographic variables and LCs as potential confounding factors. Using NHIS information, household income level was divided into 5 quintiles based on the insurance fee imposed on each household. The households in the Medical Aid program (MAP), which is a public assistance program for low-income households, were classed into the first quintile. Insurance status was classified into the MAP group and the NHIS group. The health status was divided into ‘major’ and ‘minor’ conditions based on the Handicapped Welfare Law in South Korea; samples that did not belong to either of these were categorized as “healthy.” Residence area was classed according to the number of residents in the area into either “urban” (>50,000 residents) or “rural” (<50,000 residents). LCs were diagnosed by medical doctors and defined according to the following diagnostic codes using the KCD-6: CIn (I63–I66), AP (I20), MI (I21, I22), hypertension (I10, I15), DM (E10–E15), RA (M05, M06), ED (N48, N49), osteoporosis (M80–M82), and obesity (E66).

### Statistical Analysis

Statistical analyses were conducted using the χ^2^ test to investigate the differences in variable factors. Univariate and multiple logistic regression analysis were performed to evaluate the independent association of LCs with periodontitis. To identify the risk factors associated with periodontitis, odds ratios (ORs) and 95% confidence intervals (CIs) were calculated. The χ^2^ test and logistic regression analysis were conducted with the SAS statistical package (version 9.2, SAS Institute, Cary, NC), and a *P* value < 0.005 was considered to indicate the presence of statistical significance.

## RESULTS

### Sociodemographic Factors Associated With Periodontitis

In this study, sex, age, household income, insurance status, health status, and area of residence were used as stratification variables. Among a total of 1,025,340 samples, 321,103 (31.3%) were diagnosed with periodontitis, consisting of 158,303 males (49.3%) and 162,800 females (50.7%). The prevalence of periodontitis increased with age up to 50 years between 2002 and 2013, peaked at 47.3% in the age group of 40 to 49 years, remained stable among people aged than 50 to 59 years, and then decreased sharply to 24.8% among those ages 60+ years. The prevalence rates were 27.2% and 35.7% among those in the first and fifth quintiles of household income, respectively, indicating that the prevalence of periodontitis increases with income. The prevalence rate of periodontitis was 4-fold higher in the NHIS group than in the MAP group (*P* < 0.001), and was lower in persons with severe disabilities (major group) than in those with moderate disabilities or who were healthy. The prevalence rate of periodontitis was higher among those residing in urban areas than in rural areas (*P* < 0.001, Table 1).

**TABLE 1:**
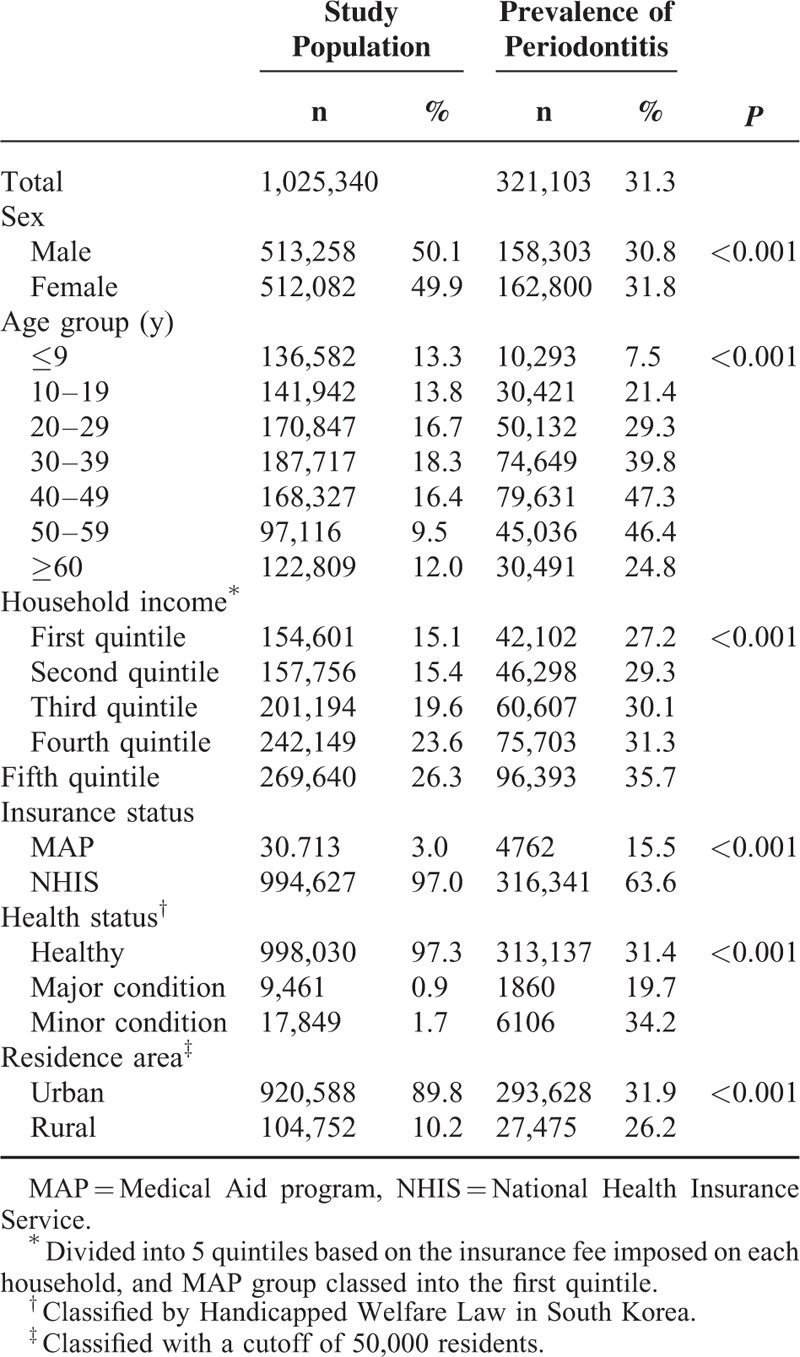
Sociodemographic Factors and Prevalence of Periodontitis in the Korean Population Included in This Study

### Comorbidities Associated with Periodontitis

LCs were detected in 551,414 (53.8%) of the samples. Among them, the prevalence rate was highest for hypertension (18.1%), followed by DM (11.6%), osteoporosis (8.6%), AP (5.7%), RA (5.6%), CIn (3.6%), MI (0.8%), obesity (0.5%), and ED (0.3%). The ‘LCs with periodontitis’ group comprised 253,538 patients and had an overall prevalence rate of 45.9%. ED had the highest prevalence rate of 55.6%, followed by AP (47.7%), RA (46.2%), DM (46.1%), osteoporosis (45.5%), MI (44.8%), hypertension (43.9%), and CIn (41.9%). These results indicate that periodontitis is strongly correlated with LCs (*P* < 0.001, Table 2).

**TABLE 2:**
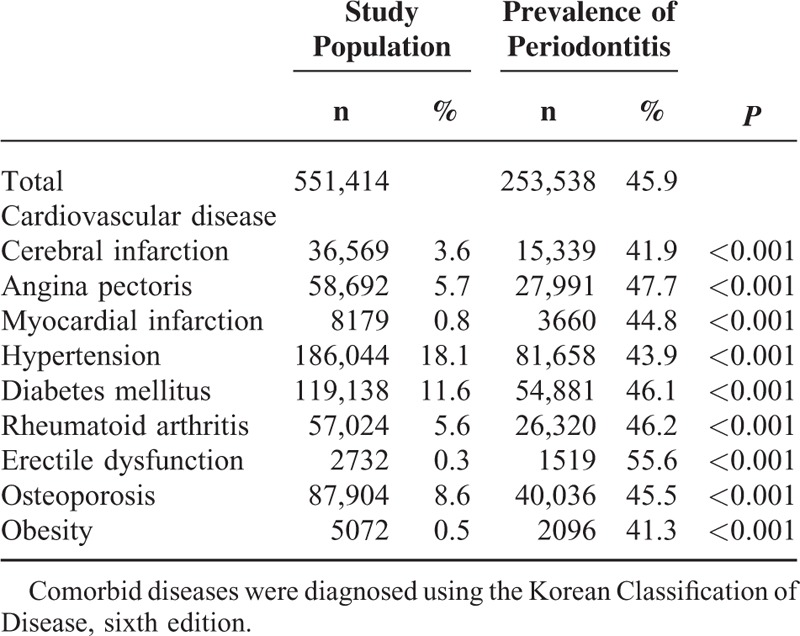
Lifestyle-Related Comorbidities and Prevalence of Periodontitis in the Korean Population Included in This Study

The overall LCs were statistical significant (*P* < 0.001) in the univariate analysis. These variables were then used in a multivariate analysis with adjusted sociodemographic and LC variables, which showed that MI was not statistically significant (OR = 0.98, 95% CI = 0.86–1.12; *P* = 0.836), whereas all of the other comorbidities were significantly related to periodontitis (Table 3).

**TABLE 3:**
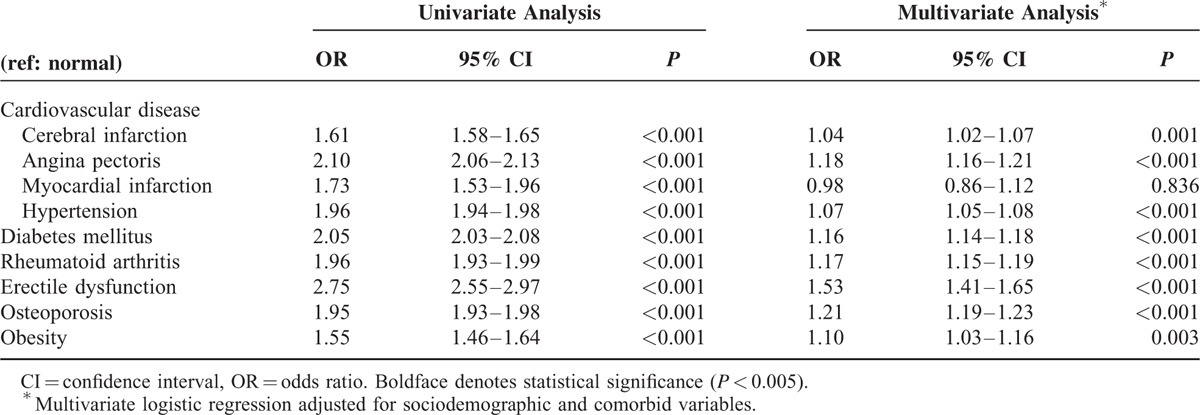
Association of Lifestyle-Related Comorbidities With Periodontitis in Univariate and Multivariate Analyses

## DISCUSSION

The recent and rapid improvements in the availability of nationwide population-based medical information have resulted in its widespread use in cohort and cross-sectional studies.^16–18^ Analysis of the KNHICD revealed that the overall prevalence rate of periodontitis was 31.3%, and was 37.5% among adults age 20+ years. This proportion is much lower than a recent study of >50% of adults being affected by periodontitis.^19^ In addition, the present study showed that the prevalence rate of periodontitis tended to be lower in those aged 60+ years and those with lower household incomes (including those in the MAP) and with greater disability (*P* < 0.001). These findings contrast with the general findings of previous studies that the rate of periodontitis is higher among those who are older and poorer.^5,20^ This discrepancy can be explained by a lower accessibility to dental services due to socioeconomic and lifestyle risk factors such as low income, low education level, poor insurance status, noncompliance with dental treatment or poor dental knowledge, constraints in physical activity, and insufficient consideration of extracted teeth due to periodontitis.^21–23^ In addition, the low prevalence rate found among those residing in rural areas in the present study is presumed to reflect the superiority of urban-based policies and medical infrastructure.^24^ The prevalence rate of periodontitis differed markedly with household income, from 27.2% among those in the first quintile to 35.7% among those in the fifth quintile (*P* < 0.001), which concurs with previous studies suggesting that health inequality is greatly affected by income.^1,25,26^

There was a high prevalence rate of LCs combined with periodontitis (41.3%–55.6%, *P* < 0.001). The univariate and multivariate analyses of the association between LCs and periodontitis showed that all variables except MI were significantly and positively related to periodontitis (*P* < 0.005). This is consistent with previous studies finding positive correlations between periodontitis and LCs.^6,16,17^

Cardiovascular disease (CVD), DM, RA, ED, osteoporosis, and periodontitis are common chronic inflammatory diseases that share similar host-related pathogeneses.^27,28^ Although CVD, DM, RA, and periodontitis are considered to share the same proinflammatory mediators, such as tumor necrosis factor-α, interleukin-1, interleukin-6, and interleukin-8, and a direct bacterial-vascular pathway, the causal relation remains unclear.^28,29^ In addition, the plasma concentration of inflammatory cytokines affects insulin resistance and type 2 DM and obesity.^30^ In the present study, obesity (OR = 1.10, 95% CI = 1.03–1.05, *P* = 0.003) was also found to be a risk factor for periodontitis, which is due to the chronic, low-grade inflammatory nature of the disease.^31^ Among 5 prospective cohort studies, it was found that the risk of CVD increased from 24% to 35% in the presence of increasing periodontitis, with a 1.14-fold increased risk (95% CI = 1.07–1.21, *P* < 0.001) of the development of CVD in patients with periodontitis.^32^ The data in the KNHICD indicated a weak negative correlation between MI and periodontitis in the present study (OR = 0.98, 95% CI = 0.86–1.12, *P* = 0.836), which contrasts with other studies showing a significant and positive correlation with periodontitis.^33,34^ These findings of the present study are due to the inclusion of subsequent MI in addition to acute MI. Subsequent MI showed a low prevalence rate, comprising only 4.9% of the entire MI population; however, in elderly patients its prevalence was higher than that of acute MI. Therefore, the reduced prevalence rate of periodontitis among those > 60 years was responsible for the multivariable analysis revealing only a weak negative correlation between periodontitis and MI.

ED and osteoporosis, with ORs exceeding 1.2, can be considered to be main risk factors among other LCs in this study.^35^ ED is a pathophysiologic factor sharing a systemic inflammation mechanism with periodontitis.^36^ It has been reported that ED might improve when periodontitis is treated^36^; however, few relevant studies have been conducted, and they have showed only a weak association between ED and periodontitis.^37^ Keller et al^16^ carried out a multivariate analysis of ED and periodontitis, which yielded an OR of 3.35. The OR was 1.53 (95% CI = 1.41–1.65, *P* < 0.001) when other confounding variables were adjusted, demonstrating a strong correlation. The database used by this study includes both acute and chronic cases; if only chronic periodontitis was investigated, it is likely that the correlations would have been stronger. The results obtained in previous studies and the present study together indicate that periodontitis is strongly associated with ED and it might be caused by the inflammatory pathway.

The prevalence rates of both periodontitis and osteoporosis increase as patients get older, and both of these diseases increase bone resorption.^38^ Osteoporosis is considered to be a risk factor for aggravating periodontitis and tooth loss, but the relationship between these 2 diseases has not been fully characterized.^39^ Most previous studies have involved small numbers of subjects, but a recent large-scale cohort study that involved 2 million people (adjusted for age) found a significant correlation between periodontitis and osteoporosis in women.^40^ The present study also found a statistically significant relationship between periodontitis and osteoporosis after adjusting variables for age (OR = 1.21, 95% CI = 1.19–1.23, *P* < 0.001). This positive correlation is considered to have resulted from a common inflammatory mechanism of the 2 diseases by the action of prostaglandins and proinflammatory cytokines.^41,42^

The current study was subject to several limitations. Firstly, this study included patients who were diagnosed with periodontitis as registered on the NHIS database and excluded patients with voluntary nonreimbursable treatment (ie, not covered by the NHIS or MAP). Secondly, the prevalence rate in this cohort was limited by the reasonably short analysis period of 11 years. Another major weakness is that smoking was not assessed by the KNHICD, which many studies have found is an important confounding factor for LCs.^5,31^ Nonetheless, the present study was strengthened by the use of complex population-based data (including >97% of the total Korean population) from the NHIS, rather than voluntary participants. The classifications in the KNHICD are based on conclusive diagnoses made by medical doctors, so the prevalence rates and associations between selected lifestyle-related diseases are highly reliable. In addition, this study considered the coefficient of variation of stratified total medical costs and used 1476 strata to ensure the representativeness of extraneous variables. The use of this precisely extracted cohort database in the current investigation of the correlation between sociodemographic and LC variables with periodontitis by adjusting confounding bias makes this a meaningful study.

## CONCLUSIONS

Significant and positive correlations were found between periodontitis and all LCs except MI using data in the KNHICD from 2002 to 2013. In particular, lifestyle-related diseases, ED, and osteoporosis are strongly correlated with periodontitis.
